# Care of patients with obesity in the Emergency Department: a joint position statement from the Brazilian Association of Emergency Medicine (ABRAMEDE) and the Brazilian Association for the Study of Obesity and Metabolic Syndrome (ABESO)

**DOI:** 10.20945/2359-4292-2024-0411

**Published:** 2025-03-11

**Authors:** Rafael Lima McGregor von Hellmann, Simone van de Sande-Lee, Maria Edna Melo, Ana Carolina Nader Vasconcelos Messias, Ian Ward Abdalla Maia, Maria Camila Lunardi, Lucas Oliveira Junqueira e Silva, Bruno Halpern

**Affiliations:** 1 Emergency Department, Monash Health, Melbourne, Australia; 2 Universidade Federal de Santa Catarina, Florianópolis, SC, Brasil; 3 Laboratório de Carboidratos e Radioimunoensaios, Hospital das Clínicas, Faculdade de Medicina, Universidade de São Paulo, São Paulo, SP, Brasil; 4 Hospital Federal dos Servidores do Estado, Rio de Janeiro, RJ, Brasil; 5 Departamento de Emergência, Hospital das Clínicas, Faculdade de Medicina, Universidade de São Paulo, São Paulo, SP, Brasil; 6 Departamento de Emergência, Hospital de Clínicas de Porto Alegre, Faculdade de MEdicina, Universidade Federal do Rio Grande do Sul, Porto Alegre, RS, Brasil; 7 Centro de Obesidade, Nove de Julho Hospital, São Paulo, SP, Brasil

**Keywords:** Obesity, Emergency medicine, Hospital Equipment and Supplies, Patient Safety

## Abstract

This document presents a joint position statement from the Brazilian Association of Emergency Medicine (ABRAMEDE) and the Brazilian Association for the Study of Obesity and Metabolic Syndrome (ABESO) regarding the management of patients with obesity in the Emergency Department. It aimed to provide recommendations for healthcare professionals and policymakers to ensure the provision of appropriate care for patients with obesity, considering their unique needs and the challenges that arise in emergency settings. The position statement addresses key issues such as the need for structural adaptations, specific equipment, and specialized training for healthcare teams. It emphasizes the complexity of emergency care for patients with obesity due to factors such as difficulties in physical examination, imaging, vascular access, and airway management. The document also discusses the prevalence of obesity, its classification, and its impact on health outcomes. It highlights the association of obesity with numerous comorbidities, including type 2 diabetes, hypertension, cardiovascular diseases, and sleep apnea. Moreover, the statement underscores the need to combat stigma and promote a supportive and respectful healthcare environment for patients with obesity. Recommendations include enhancing Emergency Department infrastructure, ensuring adequate training for professionals, and implementing public policies that support the management of obesity and its comorbidities in emergency settings.

## INTRODUCTION

This document aimed to address the particularities of caring for patients living with obesity in the Emergency Department (ED) and to present the joint position statement of the Brazilian Association of Emergency Medicine (ABRAMEDE) and the Brazilian Association for the Study of Obesity and Metabolic Syndrome (ABESO). The proposal is to provide guidelines for managers and health professionals, assisting in adapting services to the specific needs of these patients and ensuring adequate training for excellent care.

Managing patients with obesity in the ED requires adaptations in terms of structure, equipment and staff training. From the use of reinforced stretchers to the need for specific technical skills for procedures such as intubation and obtaining venous access, care involves unique challenges, especially in emergencies. The lack of adequate preparation in many units can result in critical delays in treatment and aggravating conditions that require rapid intervention.

## OBESITY: DEFINITION, EPIDEMIOLOGY AND IMPACT ON HEALTH

Since the 1980s, while malnutrition has decreased, the prevalence of obesity has increased significantly, especially among cases of severe obesity. This increase is more pronounced among populations with less access to health care, such as black and low-income people. Thus, although all patients should receive adequate treatment, the highest concentration of these patients is observed in the Unified Health System (SUS, acronym from the Portuguese *Sistema único de Saúde*).

The World Health Organization (WHO) defines obesity as the abnormal or excessive accumulation of body fat that presents a health risk (^1^). The diagnosis and classification of obesity is traditionally based on the body mass index (BMI), calculated by dividing weight in kilograms by height in meters squared. **Table 1** shows the classification of nutritional status proposed by the International Federation for the Surgery of Obesity and Metabolic Disorders (IFSO), which expands on the WHO system by including additional categories for patients with a BMI ≥ 40 kg/m^2^, without the use of terms such as “morbid” or “super-obesity”. Patients with a BMI above 50 kg/m^2^ face significantly higher risks of morbidity, mortality and reduced quality of life when compared to those with a BMI between 40 and 50 kg/m^2^ (^2^).

**Table 1:** Classification of nutritional status in adults proposed by International Federation for the Surgery of Obesity and Metabolic Disorders

BMI (kg/m^2^)	Classification
< 18,5	Underweight
18,5-24,9	Normal range
25-29,9	Overweight
30-34,9	Class I obesity
35-39,9	Class II obesity
40-49,9	Class III obesity
50-59,9	Class IV obesity
≥60	Class V obesity

BMI: body mass index.

The prevalence of obesity has increased rapidly in recent decades, both in Brazil and worldwide. According to the World Obesity Federation, in 2020, there were 2.2 billion adults (42%) living with overweight or obesity, and this figure is projected to reach 3.3 billion (54%) by 2035. Compared to 1975 data, the global prevalence of obesity has more than tripled (^3^). In Brazilian state capitals, the *Sistema de Vigilância de Fatores de Risco e Proteção para Doenças Crônicas por Inquérito Telefônico* (Vigitel) 2023 project revealed that 61.4% of the population experience overweight, while 24.3% have obesity (^4^). The 2019 National Health Survey presents even more alarming figures: 60.3% of Brazilian adults live with overweight and 25.9% with obesity (^5^). It is estimated that the proportion of people with a BMI > 35 kg/m^2^ will increase from 5.7% to 9.3% between 2019 and 2030 (^6^). In the SUS, between 2018 and 2023, the highest percentage growth occurred among individuals with class III obesity, from 2.9% to 4.4%, an increase of 52% over this period (^6^). Given this worrying scenario, it is urgent that health services prepare themselves adequately to deal with this growing profile of patients.

Despite its limitations, BMI is widely considered to be the most helpful measure for assessing obesity at a population level. However, at an individual level, it should be used as a screening tool, complemented by a more comprehensive clinical assessment. The accumulation of intra-abdominal fat is associated with a high risk of obesity-related diseases, making waist circumference a simple and practical method for identifying patients with overweight at greater risk of comorbidities due to the distribution of abdominal fat (^1^). Recently, international initiatives have proposed new diagnostic definitions with broader criteria that consider the presence of complications or functional and psychological impairment (^7^).

Obesity is a chronic, multisystem disease associated with more than 200 comorbidities, including type 2 diabetes, hypertension, dyslipidemia, cardiovascular and cerebrovascular diseases, steatotic liver disease, osteoarthritis, sleep apnea, infertility, mental disorders and various types of cancer (^8^). According to the 2024 Global Burden of Disease study, it is estimated that around 42 million deaths a year are caused by chronic non-communicable diseases (NCDs), two-thirds of which are related to overweight and obesity, including neoplasms, coronary heart disease, stroke and diabetes. In addition, the estimated number of years of healthy life lost due to NCDs is 1.6 billion (^3^).

## TRAINING AND EDUCATION

The adaptation and incorporation of obesity treatment into SUS is a challenge that must also be addressed to ensure that continuous and comprehensive support for the disease is available universally.

## CHALLENGES IN THE EMERGENCY DEPARTMENT

### Physical examination

The increase in obesity compromises traditional physical examination methods, such as inspection, palpation, auscultation and percussion, due to excess adipose tissue. Medical training, however, has not kept pace with this change, leaving students and residents unprepared to adapt to these examinations. Illustrations in academic texts often do not reflect the reality of patients with obesity, and stigma contributes to the lack of specific training (^9^).

In critically ill patients in the ED, obesity can make it challenging to identify key clinical signs. For example, in unconscious patients, the detection of a carotid or femoral pulse is fundamental in the scenario of cardiopulmonary arrest; this simple identification in individuals with severe obesity can delay adequate resuscitation of this patient (^10^). In addition, pulmonary, abdominal and gynecological examinations are particularly challenging in patients with obesity, requiring specific techniques and maneuvers to maximize the effectiveness of the physical examination (^11^).

Given the high prevalence of obesity, especially in Brazil, where more than 50% of the population has overweight, it is essential that medical schools reinforce the importance of adapting the physical examination (^5^). The teaching of these specific skills should include practice with standard patients with obesity, ensuring that future professionals are prepared to offer adequate care to this growing portion of the population (^11^).

The patient’s weight should be obtained as close to admission as possible, in a private place. This measurement is necessary for calculating the dose of medication and for the safe care of patients with obesity. The ED should have a high-capacity scale (at least 300 kg) that can accommodate patients with obesity. (^12^)

### Blood pressure measurement

For blood pressure (BP) measurement, an appropriately sized cuff is essential, so that the bladder wraps around 75 to 100% of the arm. If the largest arm BP cuff available does not fit the patient’s arm, a thigh cuff (extra large) on the upper arm can be used as an alternative. However, studies on the validity of using an extra-large cuff to measure BP in adults with obesity are limited. If this is not possible, BP can be measured on the wrist. A meta-analysis including adults with obesity showed a sensitivity of 97% and specificity of 85% for identifying hypertension when BP measured in the upper arm was compared with intra-arterial measurements. Of the alternative sites, measurements on the wrist had better sensitivity and specificity for diagnosing hypertension compared to measurements taken on the forearm or finger. When the shape of the arm is conical, ideally, a cone-shaped cuff should be used. The traditional auscultatory method, listening for Korotkoff sounds over the radial artery, should be used when it is not possible to measure BP over the brachial artery (^13^).

### Pulse oximetry

The accuracy of pulse oximetry can be affected by various conditions, such as hypothermia and vasoconstriction, and can underestimate or overestimate arterial oxygen saturation. One study evaluated the accuracy of pulse oximetry in patients with obesity in the preoperative period of bariatric surgery, comparing pulse oximetry values with arterial blood gas. Pulse oximetry overestimated oxygen saturation values in 91% of patients with a bias of 2.05%. The discrepancy was greater in patients with a BMI ≥ 40 kg/m^2^, age ≥ 40 years and a higher obesity surgery mortality risk score (^14^).

### Imaging tests

In the ED, point-of-care ultrasound (PoCUS) plays an important role in the approach to the critically ill patient (^15^). However, performing this exam in patients with obesity presents technical challenges. The hypoechogenicity of adipose tissue and the greater distance between the skin and the target organs make it difficult to obtain clear images, making assessment in emergency situations even more difficult (^16^).

In addition, due to the size of the patient, complete visualization of certain areas of the body may not be possible with a single X-ray. Examinations such as chest X-rays, for example, can be difficult to interpret due to overlapping tissues. Computed tomography (CT) and magnetic resonance imaging (MRI) devices also have limitations in terms of the size and weight they can support, which often requires multiple scans. This prolongs the examination time, increases radiation exposure and raises the risk of motion artifacts (^17^).

Currently, there are no clear guidelines on which imaging exam should be prioritized in patients with obesity in the ED. The choice usually depends on the professional’s experience and the availability of equipment.

## EMERGENCY PROCEDURES

### Vascular access

Securing venous access in emergencies is a priority, but it can be challenging, requiring multiple attempts and increasing the risk of thrombosis and infections. The antecubital fossa veins are preferred for cannulation, using a sphygmomanometer rather than a tourniquet in patients with obesity (^18^).

When peripheral venous access is not possible, central venous access is considered. However, obesity, with its excess adipose tissue, complicates the identification of the usual anatomical landmarks and is an independent factor for difficulty in venous access (^19^,^20^). As in patients with class III obesity, intraosseous access can be more difficult (^21^). The use of ultrasound is crucial to increase the success of obtaining venous access, both peripheral and central, reducing time and the risk of complications. In patients with obesity, the insertion of long peripheral cannulas should be the first option (^22^).

#### Airway management

Intubation in patients with obesity in the ED presents significant challenges. Studies suggest that although obesity increases the difficulty of intubation, other factors, such as neck circumference and Mallampati score, are more predictive of difficult intubations (^23^-^26^). In addition, although obesity seems to be associated with greater difficulty in intubation, class III obesity (BMI ≥ 40) does not seem to be associated with a significant increase in complications compared to class I and II obesity (BMI 30 to 39.9) (^24^).

Yakushiji et al. also showed that, in the ED, patients with obesity had a lower success rate in the first intubation attempt and a higher risk of adverse events (^25^).

**Figure 1:**
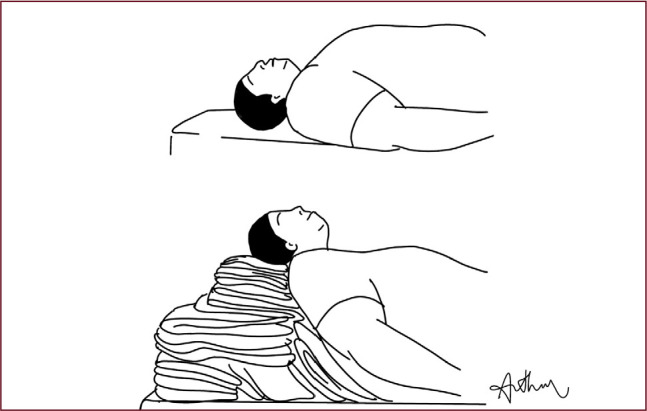
Illustrative image of ramp positioning for patients with obesity during orotracheal intubation.

Source: courtesy of Dr. Arthur de Campos Soares

The ramped position is crucial for the intubation of patients with obesity, offering important benefits such as improved laryngeal visualization and ease of intubation **(Figure 1)**. This position, achieved by elevating the head, neck and shoulders to align the external auditory meatus with the sternal notch, has been shown to significantly improve laryngeal visualization compared to the standard sniffing position. Collins et al. and Cattano et al. demonstrated better laryngeal visibility and easier ventilation in the ramped position (^27^,^28^). In addition, this position increases functional residual capacity, improving pre-oxygenation and prolonging the safe period of apnea during intubation, as reported by Dixon et al (^29^). The American Heart Association also recommends the use of the ramped position for patients with severe obesity, ensuring a safer intubation process (^30^).

Non-invasive ventilation (NIV) is effective for pre-oxygenation in patients with obesity prior to intubation, improving arterial oxygenation compared to conventional methods. Studies by Futier et al. and Delay et al. have shown that non-invasive positive pressure ventilation (NIPPV) increases partial pressure of oxygen (PaO₂) levels and oxygen concentration at the end of expiration more effectively than spontaneous breathing of 100% oxygen (^31^,^32^). However, a post-hoc analysis by Rodriguez et al. found no significant difference in the occurrence of severe hypoxemia during intubation between non-invasive positive pressure ventilation (NPPV) and high-flow nasal cannula (HFNC) in patients with acute hypoxemic respiratory failure, suggesting that HFNC may be a viable alternative in certain cases (^33^).

#### Cardiopulmonary resuscitation

The effectiveness of cardiopulmonary resuscitation (CPR) depends on early defibrillation and the quality of chest compressions (^34^,^35^). In patients with obesity, CPR follows the same guidelines as for adults with normal weight: compressions at a rate of 100 to 120 compressions per minute, with a depth of 5 to 6 cm, in a 30:2 sequence of compressions and ventilations.

However, the size and distribution of adipose tissue in patients with obesity can compromise the effectiveness of compressions. The presence of panniculus adiposus on the anterior and posterior chest wall can reduce the efficiency of compressions (^36^). Abdominal fat displaces the diaphragm upwards, similar to what happens in pregnant women, suggesting that compressions should be performed on the upper part of the sternum in patients with obesity (^37^). Studies indicate that in these patients, the maximum diameter point of the left ventricle is higher than the usual sternal compression site (^38^).

Cardiopulmonary resuscitation on patients with class III obesity or above is more tiring for rescuers, which can lead to less effective compressions (^39^). It is therefore recommended that operators take turns at shorter intervals than the standard 2 minutes. In addition, moving the patient from the bed to the floor is not necessary and can delay CPR, increasing the risk of injury (^40^).

The use of mechanical chest compression devices can be considered, although the body dimensions of patients with obesity limit the usability of these devices (^41^).

Patients with obesity have higher transthoracic impedance due to adipose tissue, but there is no evidence that BMI affects defibrillation success at the first shock. Defibrillation should start with 200 J, and modern biphasic defibrillators can compensate for the increased impedance (^42^).

### Trauma

Patients with obesity have specific pathophysiological characteristics in trauma: injuries to the limbs, pelvis and thorax are predominant, while abdominal and head trauma occur less frequently (^43^).

In traffic accidents, obesity partially protects against abdominal injuries, but increases the risk of fractures in the pelvic ring and extremities. Even low-energy trauma can result in comminuted fractures and severe skin and soft tissue injuries (^44^).

Motor, sensory and reflex assessment is challenging due to body size, impaired pain perception and reduced joint mobility. Assessment of the entire body surface, especially the perineum and lower abdomen, is essential (^44^).

Transporting patients with obesity can be extremely difficult, requiring specialized resources and appropriate equipment, which is often not available. A recent survey emphasized the need for specific training to deal with the most common challenges: extraction, transport, and the lack of aids such as cervical collars and appropriate spinal boards (^43^).

The interaction between body mass and outcome has not yet been fully explored, and the evidence in the literature is contradictory. Obesity has been associated with increased mortality following vehicle collisions, despite the severity of injuries being comparable or lower among patients with obesity (^45^). These patients are at greater risk of post-traumatic complications, including pulmonary embolism, prolonged mechanical ventilation, infections, decubitus ulcers and multiple organ failure (^46^,^47^).

Resuscitation in patients with obesity is often inadequate, with higher mortality from persistent hemorrhagic shock due to relative hypovolemia and underestimation of volume requirements (^48^,^49^).

### Medications

Medication dosage in patients with obesity is complex, as most recommendations have been extrapolated from patients without obesity, leading to dosage errors. Lipophilicity is crucial: lipophilic drugs should be dosed by total weight, while hydrophilic drugs should be dosed by ideal or adjusted weight. For drugs eliminated by the kidneys, the actual creatinine clearance should be used (^50^).

Due to pharmacokinetic and pharmacodynamic differences, drug dosing in individuals with obesity should consider ideal body weight, lean body mass, adjusted body weight or total body weight (^51^,^52^).

Inadequate drug dosing during rapid sequence intubation (RSI) in patients with obesity can increase discomfort and complications (^53^). Studies indicate that these patients often receive insufficient doses of sedatives and paralytics, such as etomidate and succinylcholine (^54^). Therefore, it is essential that emergency physicians adjust dosage according to body weight, as illustrated in **table 2** (^55^,^56^).

**Table 2:** Drug dosage for intubation in patients with obesity. Lean body weight is calculated from the formula by Janmahasatian et al.

Medication	Reference teight	Dose
Succinylcholine	Total body weight	1-1.5 mg/kg
Rocuronium	Lean body weight or ideal body weight	1-1.5 mg/kg (maximum of 250 mg)
Ketamine	Total body weight (class I and II obesity) Ideal body weight (class ≥ III)	1-2 mg/kg
Etomidate	Total body weight	0.2-0.3 mg/kg
Propofol	Lean body weight or lean body weight	1.5-2 mg/kg
Midazolam	Ideal body weight	0.1-0.2 mg/kg

Source: Janmahasatian et al. (^57^)

#### Cardiovascular drugs

Beta-blockers, digoxin and procainamide, because they are hydrophilic, should be dosed according to ideal weight. Calcium channel blockers, being lipophilic, should be dosed by total weight. Vasopressors such as norepinephrine do not require adjustment in individuals with obesity (^18^). Doses of adrenaline may also be inappropriate. Doses greater than 1 mg increase the rate of return to spontaneous circulation, but can worsen neurological outcomes (^58^,^59^).

#### Antimicrobial drugs

Obesity is associated with a pro-inflammatory state that increases the risk of nosocomial infections and organ dysfunction (^60^,^61^). Active surveillance and prudent use of antibiotics are essential to prevent multidrug-resistant infections.

The correct dosage of antimicrobials in patients with obesity is crucial, especially in sepsis (^62^). Vancomycin should be dosed by total weight, while penicillins, cephalosporins and carbapenems by the upper limit of the recommendations (^63^). Aminoglycosides should be dosed by ideal weight, or adjusted if total weight exceeds 130% of the ideal (^64^).

#### Anticoagulant drugs

The metabolic syndrome in patients with obesity induces a state of hypercoagulability, requiring careful prevention of thromboembolic events (^65^,^66^). Mechanical devices often do not adjust properly, and higher doses of low molecular weight heparin (LMWH) may be necessary.

Low molecular weight heparin is dosed at 1 mg/kg/day, but may require anti-Xa monitoring when there is severe obesity (^67^). If LMWH is not available, unfractionated heparin is an alternative (^68^). The use of new direct oral anticoagulants in patients with obesity still lacks robust studies, and consultation with a pharmacist for dosing is recommended.

## EQUIPMENT AND INFRASTRUCTURE

The equipment for the care of patients with bariatric needs must be suitable to accommodate the patient’s dimensions and weight, and pass through doors and spaces where the equipment will be used. They should be labeled in a way that facilitates easy visualization and identification of weight capacity by healthcare staff, without being stigmatizing for the patient. This equipment includes: fixed stretchers, transport stretchers, beds, pressure reduction mattresses, bariatric walkers (71-102 cm), chairs, wheelchairs, appropriately sized hospital gowns, mechanical lifts, and slings. It is essential that the entire team involved is knowledgeable and competent in safely mobilizing and transferring patients with bariatric care needs (^12^,^69^).

According to the accreditation/enabling standards for high-complexity care services for individuals with obesity, defined in Annex II of Ordinance No. 425 of the Ministry of Health, dated March 19, 2013, the equipment must have a weight capacity greater than 230 kg. The hospital bed must be a special Fowler type, electronically controlled, with Trendelemburg movement (operable via motor or crank) and a high-density mattress (^70^).

All hospital spaces used by patients with bariatric needs must be adapted to allow for the transit and accommodation of both the patients and staff. The doors (including elevator doors) must have a minimum width of 122 cm. Bariatric toilets must be floor-mounted and suitable for the patient’s weight and dimensions, as should the toilet seats. The space around the toilet should accommodate one staff member on each patient’s side. Alternatively, a hygienic bath chair may be used. The size of the room and bathroom must be appropriate, allowing access for wheelchairs, with a minimum turning radius of 180 cm, and support bars should be installed (^12^,^69^).

### Healthcare professionals

The training of healthcare professionals in obesity management involves different stages. The starting point of treatment is the correct diagnosis. Recently, the European Association for the Study of Obesity (EASO) published a new consensus for diagnosing obesity that considers not only the BMI, which has already been consolidated in various publications, but also body composition assessed by different methods, emphasizing visceral fat accumulation as the main risk factor for developing comorbidities associated with obesity (^71^). The recognition that BMI alone is insufficient for an adequate diagnosis is associated with the fact that obesity is a chronic and progressive disease, and its severity is mainly linked to visceral fat accumulation, even with a lower BMI, leading to various associated complications. As a chronic disease, new concepts are also necessary for adequately managing treatment goals.

In 2022, ABESO published a new proposal for following up with patients with obesity that considers the percentage of weight lost and sustained based on the patient’s maximum weight, creating the concepts of ‘reduced obesity’ for those with a weight loss of 5 to 10% (if initial BMI is 30 to 40 kg/m^2^) or between 10 and 15% (if initial BMI is 40 to 50 kg/m^2^); and ‘controlled obesity’ for those who maintain a loss greater than 10% (if initial BMI is 30 to 40 kg/m^2^) or greater than 15% (if initial BMI is 40 to 50 kg/m^2^) of their maximum body weight (^72^). The new proposal introduces more realistic and appropriate concepts for tracking these patients and shifts the paradigm of BMI normalization as the treatment goal.

Treating obesity as a chronic, progressive, and recurrent disease associated with comorbidities that impact morbidity and mortality is not only a paradigm shift but also the first step towards incorporating concepts aimed at reducing the stigma that prevents these patients from seeking healthcare services.

A digital survey conducted by ABESO in 2022 on obesity and weight stigma revealed that over 80% of participants, with an average BMI of around 36 kg/m^2^, had experienced some form of embarrassment related to their weight. Among those with a BMI over 40 kg/m^2^, this percentage exceeded 98%. An extremely important finding is that more than 60% of participants reported experiencing embarrassment during interactions with healthcare professionals, highlighting the importance of adequate training for the care of these patients (^73^).

One of the ways to combat the stigma associated with diseases is by using person-first language, which avoids defining an individual based on their condition. Therefore, it is recommended to use terms like “person with obesity” instead of “obese.” This language is now adopted by leading scientific journals in the field of obesity, as well as national and international organizations (^74^).

The treatment of patients with obesity begins with the appropriate approach by healthcare professionals, who need to be trained not only in the use of proper language but also in demonstrating empathy, emotional support, and positive guidance regarding potential interventions and treatment expectations during consultations. The consultation should take place in environments equipped with the necessary apparatus adapted to accommodate and examine patients with obesity (^75^).

#### For patients

Raising awareness about the chronic nature and progression of obesity is essential for educating patients on long-term weight control and maintenance. The ABESO survey showed that up to 93% of patients had previously attempted to lose weight, most of them seeking assistance in the private sector, while one-third of these patients had already tried to lose weight on their own (^76^). Concepts such as reduced and controlled obesity are very important to align the expectations between healthcare teams and patients, within a perspective of improving health and reducing long-term morbidity and mortality, compatible with the treatments available (^72^).

## PUBLIC POLICY RECOMMENDATIONS

### Institutional policies to support the care of patients with obesity

- Incorporate specific training on obesity and its comorbidities into the curricula of Emergency Medicine residency programs.
- Ensure compliance with the guidelines established in Annex II of Ordinance No. 425 of the Ministry of Health, which mandates 24-hour emergency care for patients with comorbidities associated with obesity, adjusting structures and internal processes to guarantee that these patients receive adequate and equitable care, in accordance with the principles of equity in the SUS.
- Include the patient’s weight in referral information to ensure that patients weighing over 150 kg are directed to services adequately equipped and structured to care for them.
- Establish standardized clinical protocols for the care of patients with severe obesity in emergency situations, addressing both physical adaptations and the psychological support required.
- Combat the stigma of obesity (weight stigma) through institutional awareness and education campaigns to reduce prejudice and ensure humanized and appropriate care.

### Emergency Department design and resource allocation

- Adapt ED infrastructure to ensure the availability of bariatric equipment such as stretchers, wheelchairs **(Figure 2)**, CT scanners, and beds **(Figure 3)** capable of supporting patients with severe obesity.
  **Figure 2:**
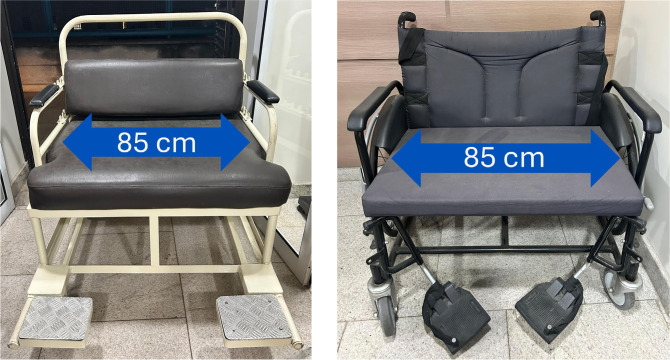
Wheelchair models for patients with obesity.

Source: courtesy of the Bariatric Surgery Unit Ward of the Digestive System Surgery Discipline of Hospital das Clínicas, Faculdade de Medicina, Universidade de São Paulo.
  **Figure 3:**
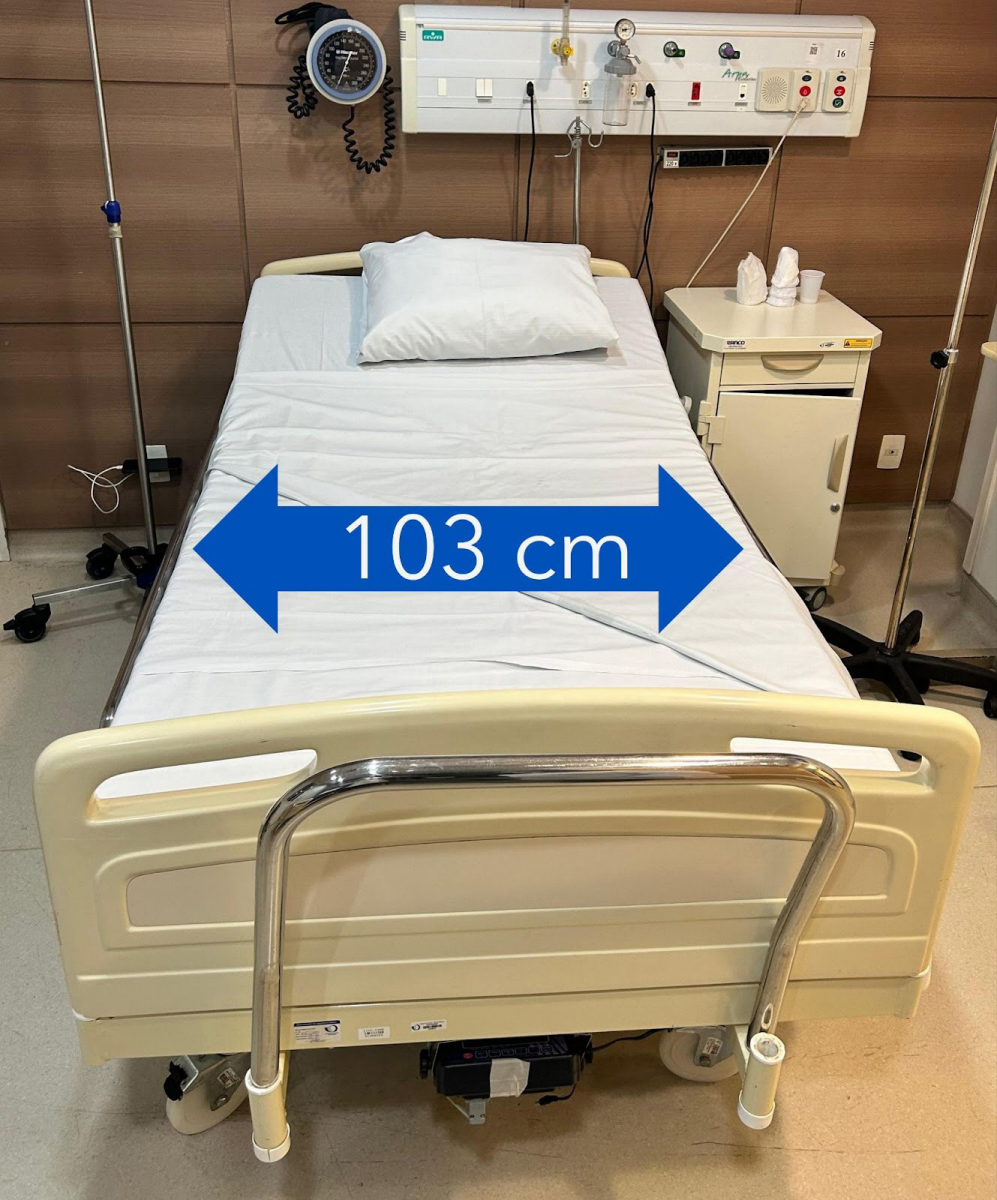
Hospital bed model for patients with obesity.

Source: courtesy of the Bariatric Surgery Unit Ward of the Digestive System Surgery Discipline of *Hospital das Clínicas, Faculdade de Medicina, Universidade de São Paulo*.
- Redesign physical spaces in EDs to ensure accessibility and safety when caring for patients with obesity, including appropriate door widths **(Figure 4**), structural reinforcement of floors, and movement areas.
  **Figure 4:**
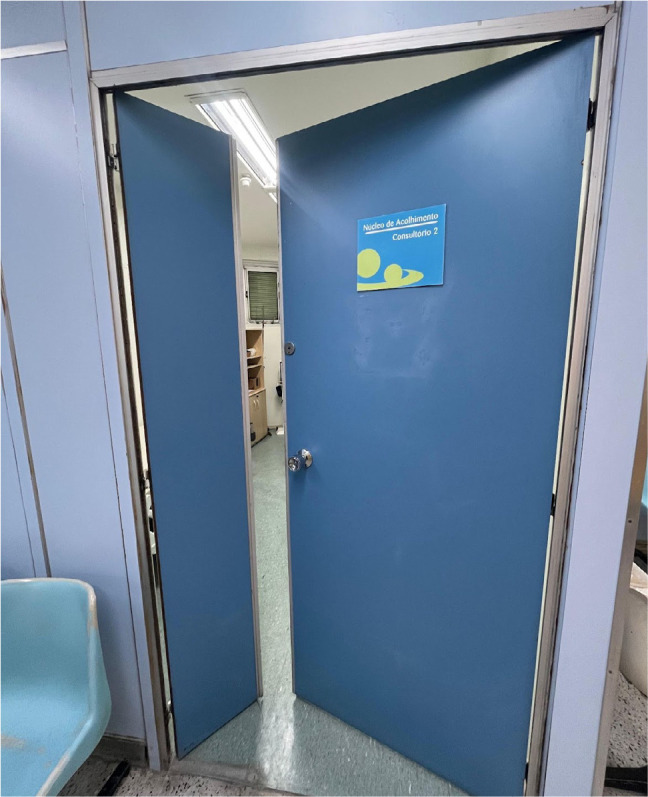
Door with opening extension for a wider stretcher and/or wheelchair.

Source: courtesy of the Digestive Digestive System Surgery Service of the *Hospital Federal dos Servidores do Estado do Rio de Janeiro*.
- Invest in monitoring and evaluation devices designed for patients with obesity, such as appropriately sized BP cuffs **(Figure 5)**, long venous cannulas, and high-capacity scales.

### Research funding and public policy changes

- Promote funding for research that explores best practices for the care of patients with obesity in emergency settings, addressing both technical specificities and the impact of stigma on care.
- Support public policies that encourage the creation of specialized centers for the treatment of patients with obesity, including interdisciplinary management programs and prevention of associated comorbidities.
- Encourage the development of new guidelines based on scientific evidence that provide practical solutions to adapt healthcare systems to the growing population with obesity, focusing on improving clinical outcomes.
- Advocate for continuous funding policies for the training of healthcare professionals in obesity management, in addition to the acquisition of appropriate equipment and the expansion of access to specialized care in the SUS.

## Conclusion

Given the rising prevalence of obesity and its associated comorbidities, it is imperative that healthcare services, particularly Emergency Departments, adapt to adequately serve this population. This involves not only appropriate infrastructure and equipment but also the continuous training of healthcare professionals to provide humanized and stigma-free care. The implementation of public policies that promote education, awareness, and the fight against weight stigma, along with the creation of specialized centers, will be crucial for improving clinical outcomes and ensuring that all patients with obesity receive the dignified and effective care they need.
